# Physical activity and physical fitness in children with heritable connective tissue disorders

**DOI:** 10.3389/fped.2023.1057070

**Published:** 2023-03-17

**Authors:** Lisanne de Koning, Jessica Warnink-Kavelaars, Marion van Rossum, Selina Limmen, Ruth Van der Looven, Laura Muiño-Mosquera, Annelies van der Hulst, Jaap Oosterlaan, Lies Rombaut, Raoul Engelbert, on behalf of the Pediatric Heritable Connective Tissue Disorders Study Group

**Affiliations:** ^1^ Center of Expertise Urban Vitality, Faculty of Health, University of Applied Sciences Amsterdam, Amsterdam, Netherlands; ^2^ Department of Rehabilitation Medicine, Amsterdam UMC Location University of Amsterdam, Amsterdam, Netherlands; ^3^ Amsterdam Movement Sciences, Rehabilitation and Development, Amsterdam, Netherlands; ^4^ Department of Pediatrics, Emma Children's Hospital, Academic Medical Center, Amsterdam, Netherlands; ^5^ Amsterdam Rheumatology and Immunology Center, Reade, Amsterdam, Netherlands; ^6^ Department of Physical and Rehabilitation Medicine, Child Rehabilitation, Ghent University Hospital, Ghent, Belgium; ^7^ Department of Pediatrics, Division of Pediatric Cardiology, Ghent University Hospital, Ghent, Belgium; ^8^ Center for Medical Genetics, Ghent University Hospital/Ghent University, Ghent, Belgium; ^9^ Department of Pediatric Cardiology, Amsterdam UMC, Location University of Amsterdam, Amsterdam, Netherlands; ^10^ Department of Pediatrics, Emma Children's Hospital Amsterdam UMC Follow-Me Program & Emma Neuroscience Group, Emma Children's Hospital, Amsterdam UMC, University of Amsterdam, Amsterdam, Netherlands; ^11^ Amsterdam Reproduction and Development Research Institute, Amsterdam, Netherlands

**Keywords:** Heritable Connective Tissue Disorders, Marfan Syndrome, Ehlers Danlos Syndromes, Loeys Dietz Syndrome, physical activity, physical fitness

## Abstract

**Objectives:**

Health problems in patients with heritable connective tissue disorders (HCTD) are diverse and complex and might lead to lower physical activity (PA) and physical fitness (PF). This study aimed to investigate the PA and PF of children with heritable connective tissue disorders (HCTD).

**Methods:**

PA was assessed using an accelerometer-based activity monitor (ActivPAL) and the mobility subscale of the Pediatric Evaluation of Disability Inventory Computer Adaptive Test (PEDI-CAT). PF was measured in terms of cardiovascular endurance using the Fitkids Treadmill Test (FTT); maximal hand grip strength, using hand grip dynamometry (HGD) as an indicator of muscle strength; and motor proficiency, using the Bruininks-Oseretsky Test of Motor Proficiency-2 (BOTMP-2).

**Results:**

A total of 56 children, with a median age of 11.6 (interquartile range [IQR], 8.8–15.8) years, diagnosed with Marfan syndrome (MFS), *n* = 37, Loeys-Dietz syndrome (LDS), *n* = 6, and genetically confirmed Ehlers-Danlos (EDS) syndromes, *n* = 13 (including classical EDS *n* = 10, vascular EDS *n* = 1, dermatosparaxis EDS *n* = 1, arthrochalasia EDS *n* = 1), participated. Regarding PA, children with HCTD were active for 4.5 (IQR 3.5–5.2) hours/day, spent 9.2 (IQR 7.6–10.4) hours/day sedentary, slept 11.2 (IQR 9.5–11.5) hours/day, and performed 8,351.7 (IQR 6,456.9–1,0484.6) steps/day. They scored below average (mean (standard deviation [SD]) *z*-score −1.4 (1.6)) on the PEDI-CAT mobility subscale. Regarding PF, children with HCTD scored well below average on the FFT (mean (SD) *z*-score −3.3 (3.2)) and below average on the HGD (mean (SD) *z*-score −1.1 (1.2)) compared to normative data. Contradictory, the BOTMP-2 score was classified as average (mean (SD) *z*-score.02 (.98)). Moderate positive correlations were found between PA and PF (r(39) = .378, *p* < .001). Moderately sized negative correlations were found between pain intensity and fatigue and time spent actively (r(35) = .408, *p* < .001 and r(24) = .395 *p* < .001, respectively).

**Conclusion:**

This study is the first to demonstrate reduced PA and PF in children with HCTD. PF was moderately positively correlated with PA and negatively correlated with pain intensity and fatigue. Reduced cardiovascular endurance, muscle strength, and deconditioning, combined with disorder-specific cardiovascular and musculoskeletal features, are hypothesized to be causal. Identifying the limitations in PA and PF provides a starting point for tailor-made interventions.

## 1. Introduction

Children with chronic disease often show physical inactivity leading to a reduction in PF level, thereby inducing a downward spiral of further physical inactivity (1). Physical activity (PA) and physical fitness (PF) have been described as important health-related outcomes in all age groups, especially for children with chronic conditions (2–5).

PF refers to the ability to perform physical activities and a full range of physiological and psychological qualities. PA can be defined as any bodily movement produced by muscle action that increases energy expenditure (5). PF is related to the components of fitness that benefit from a physically active lifestyle, including three main components: cardiorespiratory fitness, muscular fitness, and speed/agility (5–7).

Health problems in children with heritable connective tissue disorders (HCTD) are diverse and complex and characterized by multisystemic involvement (8–11). The phenotypes of the most common HCTD, Marfan syndrome (MFS) (9), Loeys-Dietz syndrome (LDS) (12) and Ehlers-Danlos syndrome (EDS) (11) show similarities in cardiovascular (aortic aneurism, mitral valve prolapse), musculoskeletal (e.g., scoliosis, foot deformities, joint hypermobility) and cutaneous features (e.g., skin hyperextensibility and tissue fragility). Children with MFS and EDS and their parents report problems with keeping up with peer activities and participation in school, sports, and other leisure activities due to fatigue, pain, and physical impairment (13–16).

To date, PA and PF have not been investigated in detail in children with HCTD. However, several studies have investigated PA and PF in children with other chronic conditions. These studies have reported significantly lower PA levels in children with juvenile idiopathic arthritis (JIA), type 1 diabetes, and obesity (17–19). More specifically, results showed that these children spent significantly less time in moderate to vigorous physical activities and more time in sedentary activities than healthy peers (17, 18, 20). In addition, children with JIA scored significantly lower on PF in terms of lower muscle strength, muscular endurance, and aerobic- and anaerobic- capacity than healthy peers (19). Children with generalized joint hypermobility (GJH) without a genetic diagnosis showed no differences in the level and duration of daily PA compared to healthy controls (21). However, another study reported a decrease in maximum exercise capacity in these children (22). It is unknown to what extent these findings translate to children with HCTD.

In general, it seems plausible that children with a higher level of PF spent more hours physically active during the day. However, the mutual relationship between PA and fitness has not been investigated recently (23). Insights into daily PA and PF in children with HCTD are mandatory to provide a starting point for tailored interventions (4). Therefore, the current study aimed to investigate PA and PF and their interrelationship in children with HCTD, specifically MFS, LDS, and genetically confirmed types of EDS.

## 2. Materials and methods

### 2.1. Study design and patient selection

This was a multicenter, observational, cross-sectional study. Participants were recruited from the Expert Center for MFS and related hereditary connective tissue disorders in Amsterdam, the Netherlands, and the Center for Medical Genetics of the Ghent University Hospital in Belgium. Eligible for inclusion were all children with HCTD, aged between 6 and 18 years, who were diagnosed with MFS (9), LDS (12) and genetically confirmed types of EDS, hereafter referred to as EDS (11). The exclusion criteria were comorbid prominent chronic diseases affecting physical functioning, PA, PF, and cognitive impairment (IQ < 80) and medical or psychiatric disorders that may affect the measurements in this study.

### 2.2. Procedure

The Medical Ethics Review Committee of the Amsterdam UMC (2019_121) and the Ethical Committee of Ghent University Hospital (EC2019/1958) approved the study protocol. The children and their parents were invited by letter. Informed consent was signed by the parents (for children aged <12 years), parents and children (for children aged 12 to 16 years), or adolescents (for participants aged ≥ 16 years). Approval from a pediatric cardiologist was required to participate in this study. All measurements were performed in a fixed order taking into account the intensity of the different tests and adequate rest pauses. Examiners were experienced with the specific tests and the standardized test protocol has been intensively trained. Tests were performed between March 2020 and March 2021.

### 2.3. Measures

#### 2.3.1. Diagnostic and socio-demographic data

Disease-related data (diagnosis and use and type of cardiovascular medication) and socio-demographic data (age and sex) were collected using a custom-made questionnaire completed by the parents.

#### 2.3.2. Clinical characteristics

The assessed clinical characteristics included body mass index (BMI) and the presence of GJH, as assessed using the Beighton scale (24).

Pain intensity over the last week was measured using a visual analog scale (VAS) (25) and scored on a 0–100 mm scale, with 0 mm referring to “no pain” and 100 mm to “very severe pain” (26). The validity and reliability of this method for assessing pain have been demonstrated in children with chronic diseases (25, 26).

Fatigue was measured using the Patient Reported Outcomes Measurement Information System (PROMIS) Fatigue 10a Pediatric v2.0 short form assessing self-reported fatigue in children 8–18 years of age or the Fatigue 10a Parent Proxy v2.0 short form assessing parent-reported fatigue in children <8 years of age. Both questionnaires demonstrated excellent psychometric properties (27, 28).

#### 2.3.3. Physical activity

##### 2.3.3.1. Accelerometry

PA was assessed using an ActivPAL™ accelerometer (Pal Technologies Ltd., Glasgow, United Kingdom). ActivPAL™ is a validated and reliable instrument for children (29, 30). The ActivPAL™ quantifies PA (standing, walking, and cycling), sedentary behavior (lying down, sitting, and passive traveling), and time spent asleep in daily life. Participants wore the ActivPAL™ for seven consecutive days during regular school weeks on the middle anterior line of the right thigh, sealed with a non-allergenic adhesive tape. The average time spent active, sedentary, sleeping, and daily steps were calculated.

##### 2.3.3.2. Daily mobility

Daily mobility was assessed using the Pediatric Evaluation of Disability Inventory Computer Adaptive Test (PEDI-CAT). The PEDI-CAT is a validated and reliable assessment tool for parents and caregivers (31, 32) reporting on the daily mobility of their children and quantifying limitations in daily activity. Only the mobility subscale was used to assess five content areas: basic movement and transfers, standing and walking, steps and inclines, running and playing, and wheelchairs (33).

#### 2.3.4. Physical fitness

##### 2.3.4.1. Cardiovascular endurance

Cardiovascular endurance was assessed using the standardized Fitkids Treadmill Test Protocol (FTT) (34). The FTT is an incremental treadmill test consisting of 90 second stages with increments in speed and grade. After a warming-up period (3.5 km/h, 0% grade), the test starts at 3.5 km/h and a 1% gradient, followed by incremental increases in speed (0.5 km/h) and incline (2%) until exhaustion (34). The time to exhaustion (TTE) was defined as the point when the participant stopped the test despite verbal encouragement minus the 1.5 minute warming-up. The FTT has good validity and reproducibility in children aged 6–18 years of age (34).

##### 2.3.4.2. Grip strength

Grip strength is a good indicator of overall muscle strength in children and adolescents (35). Grip strength was measured using the Biometrics E-Link Evaluation System (Biometrics Ltd., Gwent, UK) with a standard handgrip dynamometer (HGD). The standardized testing position recommended by the American Society of Hand Therapists (ASHT) to measure grip strength was used (36). The maximal grip strength was recorded in kilograms of force as the mean of three successive trials. The Biometrics E-Link Evaluation System is a validated and reliable instrument for assessing grip strength (37).

##### 2.3.4.3. Motor proficiency

The Bruininks-Oseretsky Test of Motor Proficiency-2 (BOTMP-2) was used to assess motor performance (38). The BOTMP-2 generates scores on four composite measures: fine manual control, manual coordination, body coordination, and strength and agility. The BOTMP-2 has been validated and is reliable for assessing motor performance in children (39).

### 2.4. Statistical analysis

Data were exported from the Castor database (Electronic Data Capture, Ciwit BV, Amsterdam, The Netherlands, 2021) to the Statistical Package for Social Science (SPSS) version 26.0.

The percentage of missing values was <15% for the PF parameters (FTT 14.3%, HGD 5.4%, BOTMP-2 3.6%, respectively). Missingness was assessed as “missing at random”. Multivariate Imputation by Chained Equations (MICE) with predictive mean matching was used to impute the missing data (40, 41). The percentage of missing values on the PA parameters (including PROMIS V2.0 Shortform fatigue, PEDI-CAT, and ActivPAL) was >15% and therefore not suitable for imputation. Therefore, complete case analysis was used for analyzing PA parameters.

The comparability of the group of children with and without data of the PROMIS V2.0 Shortform fatigue, PEDI-CAT, and ActivPAL was assessed for age and sex using Chi-square tests and t-tests. The sample characteristics, age, and sex of the total HCTD group were comparable for the complete cases on the questionnaires and activity assessments (PROMIS V2.0 Shortform fatigue, PEDI-CAT, and ActivPAL, respectively) and proved to be representative.

Data of the total HCTD group and the MFS, LDS, and EDS subgroups are described in terms of mean and standard deviations (SD) or median and interquartile range (IQR) when indicated. Age- and sex-adjusted normative data were available for all measures (PEDI-CAT (42) FTT (34), HHD (43) and BOT-MP 2 (38)) except for ActivPAL. Data were converted to z-scores for the HCTD group and the MFS, LDS, and EDS subgroups. *Z*-scores were interpreted as well above average (z-score: ≥ 2), above average (*z*-score between 1 and 2), average (z-score between 1 and −1), below average (z-score between −1 and −2), and well below average (z-score ≤ −2). ActivPAL data were not converted to z-scores.

The exploratory analysis compared the PF data of children using and not using cardiovascular medication and children with and without GJH (Beighton score ≥6 yes/no). Z-scores between subgroups were compared using the t-test. Pearson's R or Spearman's rho was used to explore the relationships between PA, PF, and clinical characteristics of pain and fatigue in the HCTD group. The magnitude of correlations was interpreted according to Cohen, distinguishing between small-sized (0.10–0.30), medium-sized (0.30–0.50), and large-sized correlations (≥0.50) (44).

## 3. Results

### 3.1. Diagnostic and socio-demographic data

Table 1 shows the diagnostic and socio-demographic data and the clinical characteristics of the participants. Fifty-six children participated, of which 37 (66%) were diagnosed with MFS, 6 (11%) with LDS, and 13 (23%) with EDS (classical EDS *n* = 10, vascular EDS *n* = 1, dermatosparaxis EDS *n* = 1, arthrochalasia EDS *n* = 1), with a median age of 11.6 (IQR 8.8–15.8) years.

**Table 1:** Patient characteristics.

	HCTD (*N* = 56)	MFS (*N* = 37)	LDS (*N* = 6)	EDS (*N* = 13)
Sex, female *N* (%)	23 (41)	16 (43)	3 (50)	4 (31)
Age (years), median (IQR)	11.6 (8.8–15.8)	10.5 (8.0–15.0)	13.6 (10.1–16.4)	14.4 (9.2–16.5)
BMI, median (IQR)	15.9 (14.2–20.7)	15.6 (14.0–16.6)	17.0 (13.9–19.1)	18.1 (17.0–21.0)
Beighton score/9, median (IQR)	3 (1–6)	2 (0–4)	3 (0–5)	6 (5–7)
GJH (Beighton ≥6), *N* (%)	16 (29)	5 (14)	1 (17)	10 (77)
**Pain intensity - VAS**
past 7 days, mean (SD)	1.3 (1.9)	1.2 (1.7)	1.3 (2.1)	1.6 (2.3)
Z-scores, mean (SD)	.66 (.94)	.61 (.86)	.63 (1.0)	.81 (1.2)
**Fatigue - Promis V2.0 shortform reported by the child**
T score, mean (SD)	41.7 (9.8)	40.7 (10.1)	41.2 (9.5)	43.9 (9.9)
Z-scores, mean (SD)	−.83 (.98)	−.88 (.95)	−.83 (.95)	−.62 (1.0)
**Fatigue - Promis V2.0 shortform reported by the parent**
T score, mean (SD)	46.5 (11.2)	44.8 (11.4)	49.3 (11.0)	49.9 (11.1)
Z-scores, mean (SD)	−.35 (1.1)	−.52 (1.1)	−.07 (.96)	−.01 (1.1)
Use of CVM, *N* (%)*	20 (36)	16 (43)	4 (67)	0 (0)
ARBs	9 (45)	7 (44)	2 (50)	
BBs	10 (50)	8 (50)	1 (25)	
Both ARBs & BBs	1 (5)	0 (0)	1 (25)	

HCTD, heritable connective tissue disorders; MFS, marfan syndrome; LDS, loeys-dietz syndrome; EDS, ehlers-danlos syndromes; VAS, visual analogue scale; GJH, generalized joint hypermobility; CVM, cardiovascular medication; ARBs, angiotensin receptor blockers; BBs, beta-blockers.

### 3.2. Clinical characteristics

Table 1 shows that the BMI was an average of 15.9 (IQR 14.2–20.7); 29% scored ≥6 on the Beighton scale, and 36% used cardiovascular medications in the HCTD group. The z-scores for pain intensity and fatigue for the HCTD group and the subgroups were in the average range compared to normative data.

### 3.3. Physical activity

PA in children with HCTD is illustrated in Table 2.

**Table 2:** Physical activity in children with HCTD.

ACTIVPALᵀᴹ	HCTD (*N* = 33)	MFS (*N* = 25)	LDS (*N* = 2)	EDS (*N* = 6)
Sedentary time in hours, median (IQR)	9.2 (7.6–10.4)	8.7 (7.4–10.1)	11.0 (9.5–n.a.)	9.4 (8.0–11.0)
Active time in hours, median (IQR)	4.5 (3.5–5.2)	4.5 (3.5–5.2)	3.2 (2.7–n.a.)	4.2 (4.0–5.2)
Hours of sleep, median (IQR)	11.2 (9.5–11.5)	11.2 (9.8–11.9)	10.3 (9.7–n.a)	10.3 (8.5–11.4)
Steps/day, median (IQR)	8351.7 (6456.9–10,484.6)	8527.9 (6779.1–10,429.6)	6575.6 (6456.6–n.a.)	8419.1 (6142.6–14,906.1)
**PEDI-CAT**	**HCTD (*N* = 41)**	**MFS (*N* = 24)**	**LDS (*N* = 4)**	**EDS (*N* = 13)**
Mobility t-score, mean (SD)	36.2 (16.1)	39.0 (15.6)	30.0 (21.7)	33.2 (15.7)
Z-score	−1.4 (1.6)	−1.1 (1.6)	−2.0 (2.2)	−1.7 (1.6)

HCTD, heritable connective tissue disorders; MFS, marfan syndrome; LDS, loeys-dietz syndrome; EDS, ehlers-danlos syndromes; PEDI-CAT, pediatric evaluation of disability inventory computer adaptive test; ActivPAL, an accelerometer-based activity monitor; n.a, not applicable due to small sample size.

#### 3.3.1. Accelerometry

ActivPAL data were available for 33 of 56 (59%) children. Fifty-five children have worn the pre-set and working ActivPAL device. One child did not want to wear the devise (*n* = 1) and one child removed the device due to itching (*n* = 1). When loading the data afterwards, it turned out that a number of devices had not registered any data (*n* = 21). Concerning the complete cases, children with HCTD were active for 4.5 h/day (median = 4.5, IQR 3.5–5.2), spent 9.2 h/day sedentary (median = 9.2, IQR 7.6–10.4 h/day), slept 11.2 h/day (median = 11.2, IQR 9.5–11.5 h/day), and performed on average 8,351.7 steps a day (median = 8,351.7, IQR 6,456.9–10,484.6 steps a day).

#### 3.3.2. Daily mobility

The PEDI-CAT mobility subscale data were available for 41 of the 56 children (73%). Missingness was due to absent of the parent during the measurement (*n* = 2) or technical issues with the software or computer (*n* = 13). Regarding the complete cases, the reported score on the mobility subscale of the PEDI-CAT was below average for the total HCTD group and subgroups compared to normative data, indicating reduced daily physical mobility in children with HCTD.

### 3.4. Physical fitness

PF parameters in children with HCTD are shown in Table 3.

**Table 3:** Physical fitness in children with HCTD.

	HCTD (*N* = 56)	MFS (*N* = 37)	LDS (*N* = 6)	EDS (*N* = 13)
**FTT**
Time to exhaustion in minutes, mean (SD)	8.9 (2.8)	8.8 (2.9)	8.3 (1.5)	9.4 (3.1)
Z-score, mean (SD)	−3.3 (3.2)	−3.0 (2.9)	−4.2 (2.6)	−3.6 (4.0)
**HGD**
dominant hand in kg, mean (SD)	18.1 (8.0)	18.2 (8.6)	17.0 (6.8)	18.3 (7.5)
Z-score, mean (SD)	−1.1 (1.2)	−.85 (1.6)	−1.7 (.57)	−1.5 (.80)
Non dominant hand in kg, mean (SD)	15.7 (7.7)	15.9 (8.4)	14.7 (5.1)	15.6 (6.9)
Z-score, mean (SD)	−1.9 (1.0)	−1.8 (1.2)	−2.2 (.55)	−2.0 (.91)
**BOTMP-2**
** Fine manual control**
Standard scores, mean (SD)	50.2 (9.8)	50.3 (10.6)	49.7 (8.3)	50.0 (8.6)
Z-score, mean (SD)	.02 (.98)	.03 (1.1)	−.02 (.83)	.00 (.86)
** Manual coordination**
Standard scores, mean (SD)	49.5 (8.5)	49.7 (9.2)	53.0 (8.6)	47.4 (5.8)
Z-Score, mean (SD)	−.05 (.85)	−.03 (.92)	.30 (.86)	−.26 (.58)
Body coordination	47.0 (9.6)	49.1 (10.2)	43.5 (6.7)	42.8 (7.1)
Standard scores, mean (SD)	−.30 (.96)	−.09 (1.02)	−.65 (.67)	−.72 (.71)
** Z-score, mean (SD)**
** Strength and agility**
Standard scores, mean (SD)	47.1 (7.7)	47.1 (8.7)	46.8 (6.1)	47.3 (5.6)
Z-score, mean (SD)	−.29 (.77)	−.29 (.84)	−.32 (.62)	−.27 (.56)
** Total test score**
Standard scores, mean (SD)	47.6 (9.1)	48.9 (10.3)	46.2 (9.1)	45.3 (5.4)
Z-score, mean (SD)	−.24 (1.0)	−.11 (1.03)	−.38 (.61)	−.53 (.54)

HCTD, heritable connective tissue disorders; MFS, marfan syndrome; LDS, loeys-dietz syndrome; EDS, ehlers-danlos syndromes; FTT, fitkids treadmill test; HGD, handgrip dynamometry; BOTMP-2, the bruininsk-oseretsky test of motor proficiency-2; kg, kilogram; N, numbers; SD, standard deviation.

#### 3.4.1. Cardiovascular endurance

Compared to normative data, the TTE on the treadmill test was well below the average for the HCTD group and the HCTD subgroups, indicating considerably reduced cardiovascular endurance in children with HCTD.

#### 3.4.2. Grip strength

The GHD of the dominant- and non-dominant hand was below average for the HCTD group and the EDS and LDS subgroups, indicating reduced muscle strength in children with HCTD.

#### 3.4.3. Motor proficiency

BOTMP-2 scores were interpreted as the average of the total score and all subscale scores for the HCTD and HCTD subgroups, indicating normal motor performance.

### 3.5. Exploratory subgroup analysis

There were no significant differences in PF between children who did or did not use cardiovascular medication (FFT *p* = 0.739, HGD *p* = 0.563, BOTMP-2 *p* = 0.750) or between children classified with and without GJH (FFT *p* = 0.825, HGD *p* = 0.439, BOTMP-2 *p* = 0.912).

### 3.6. Relationship between physical activity and physical fitness

PA (daily mobility) and PF and cardiovascular endurance were moderately positively correlated (r(39) = 0.378, *p* ≤ .001). The clinical characteristics of pain intensity and fatigue were moderately sized and negatively correlated with the time spent actively (r(35) = 0.408, *p* ≤ .001, r(24) = 0.395, *p* ≤ .001, respectively).

## 4. Discussion

This is the first study to investigate PA and PF in children with HCTD. We found that children with HCTD have less than average cardiovascular endurance, below-average grip strength as an indicator of total muscle strength, normal motor proficiency, and below-average mobility in daily PA compared to normative data. PA and PF were moderately correlated.

Our findings regarding PF agree with previous research on children with various chronic conditions, such as JIA and diabetes (17, 18, 20). However, several disorder-specific clinical characteristics, including cardiovascular and musculoskeletal systems, may lead to decreased daily PA and PF in children with HCTD.

For PA, we measured daily activity and mobility. Our results on daily activity were comparable with those of studies in children with other chronic conditions, such as JIA, measured using questionnaires and diaries. They concluded that children with JIA had lower PA, spent less time in moderate to vigorous PA, and spent more time sedentary than healthy controls (20, 45). Other studies have indicated that children with or without a chronic condition have lower PA levels than those recommended in exercise guidelines (46).

In addition to measuring the quantity of activities, it is also important to provide information about the ability to perform activities that are important in daily life (daily mobility). A previously conducted qualitative study of adolescents with HCTD and their parents reported that they experienced difficulties performing everyday activities related to leisure activities and sports (13, 14, 16). These observations are consistent with the below-average mobility on the PEDI-CAT mobility subscale in our study group.

Regarding PF, children with MFS and LDS are commonly diagnosed with aortic dilatation, regardless of whether it is combined with mitral valve regurgitation (8, 47). This may affect PF. We investigated whether cardiac involvement contributed to cardiovascular endurance. Almost 40% of the children in our population used cardiovascular medications (angiotensin receptor blockers [ARBs] or beta-blockers [BB]), which are only prescribed in cases of evident aortic root dilatation (48). However, we did not find significant differences in cardiovascular endurance between children with and without cardiovascular medication. The effect of medication and its relation to cardiovascular endurance have been studied in various adult populations, but the findings are contradictory (49, 50). Additionally, children with EDS did not use cardiovascular medication, and their cardiovascular endurance was in line with that of the other subgroups.

In addition to the cardiovascular system, some musculoskeletal features may also affect cardiovascular endurance and muscle strength. GJH is present in approximately 30% of our population and has been previously linked to lower PF (51). In contrast, in line with more recent studies (52, 53), we did not find significant differences between children with and without GJH. Contributing factors, such as pain, fatigue, multi-systemic dysfunction, loss of postural control, and pain-related fear, may cause decreased muscle strength, motor performance, and lower PA levels in adolescents and adults with chronic musculoskeletal pain, with or without GJH (52, 54–56). Features related to decreased exercise capacity and muscle weakness, such as scoliosis and anterior chest deformation, are frequently reported in children with HCTD (8) and other populations (51, 57, 58).

There is an international trend to promote PA in children with and without chronic conditions. The general advice is to stimulate all children to be more physically active, resulting in beneficial health effects (59, 60). There is a need for interventions to address the limitations in PF and create optimal conditions for increasing daily activity and beneficial health effects in children with HCTD. However, specific advice regarding aortic status should be considered (61).

Our results should be viewed within the limitations of the present study. First, due to mainly technical problems, there were missing data on ActivPAL and PEDI-CAT. The reasons for missing data were beyond the influence of the assessors or researchers and labelled as missing at random. However, the samples without ActivPAL and PEDI-CAT data were still comparable to the HCTD group in terms of sex and age distribution and were, therefore, representative. Second, ActivPAL data were collected during the COVID-19 pandemic. There are indications that Dutch children had significantly lower PA and higher sedentary screen time during the pandemic (62, 63). Nevertheless, our results agree with those of studies conducted on children with JIA before the COVID-19 pandemic (20, 45). Third, we could not perform statistical tests to compare the MFS, LDS, and EDS subgroups because of the small sample size.

In conclusion, this study is the first to demonstrate reduced PA and PF in children with HCTD. PA and PF were moderately positively correlated in children with HCTD. Reduced cardiovascular endurance, muscle strength, and deconditioning combined with disorder-specific cardiovascular and musculoskeletal features are hypothesized to be causal. Identifying the limitations in PA and PF provides a starting point for tailor-made interventions.

## Data Availability

The datasets generated for this study are available on request to the corresponding author.

## The Pediatric Heritable Connective Tissue Disorders Study Group

Marieke J. H. Baars (University of Amsterdam, Amsterdam); Eelco Dulfer (University Medical Center Groningen, Groningen); Yvonne Hilhorst-Hofstee (Leiden University Medical Center, Leiden); Marlies J. E. Kempers (Radboud University Medical Center, Nijmegen); Ingrid P. C. Krapels (Maastricht University Medical Center, Maastricht); Bart L. Loeys (Radboud University Medical Center, Nijmegen; University of Antwerp and Antwerp University Hospital, Antwerp); and Femke Stoelinga (University of Amsterdam, Amsterdam; Rehabilitation and Development, Amsterdam).
